# Implementing a universal informed consent process for the *All of Us* Research Program

**Published:** 2019

**Authors:** Megan Doerr, Shira Grayson, Sarah Moore, Christine Suver, John Wilbanks, Jennifer Wagner

**Affiliations:** 1 Sage Bionetworks, 2901 Third Avenue Seattle, WA 98121, USA, megan.doerr@sagebionetworks.org; 2 Center for Translational Bioethics & Health Care Policy, Geisinger, 100 N. Academy Ave., MC 30-42, Danville, PA 17822, USA

**Keywords:** Informed consent, conflicts of law, choice of law, ELSI, bioethics

## Abstract

The United States’ *All of Us* Research Program is a longitudinal research initiative with ambitious national recruitment goals, including of populations traditionally underrepresented in biomedical research, many of whom have high geographic mobility. The program has a distributed infrastructure, with key programmatic resources spread across the US. Given its planned duration and geographic reach both in terms of recruitment and programmatic resources, a diversity of state and territory laws might apply to the program over time as well as to the determination of participants’ rights. Here we present a listing and discussion of state and territory guidance and regulation of specific relevance to the program, and our approach to their incorporation within the program’s informed consent processes.

## 1. Background

### 1.1 *The* All of Us *Research Program*

The *All of Us* Research Program (AoURP) is a longitudinal national cohort program funded by the United States (US) National Institutes of Health (NIH) with investigators, study infrastructure, data management systems, and governance schema distributed across the US. All participating institutions signed Reliance Agreements ceding authority to the *All of Us* Institutional Review Board (AoU IRB) for ethical and regulatory oversight.

AoURP aims to enroll one million or more persons living within the US to contribute personal health information, including protected health information and biospecimens, to a central resource designed to accelerate research and improve health. Recruitment goals were established based on US 2040 census projections with purposeful oversampling of populations traditionally underrepresented in biomedical research to ensure sufficient statistical power for subpopulation analysis. The program intends to follow participants for at least 10 years.

Germane to AoURP is the well-documented geographic mobility of the US population, with the percentage of those living in the US who report having moved in the past 5 years at least 2 times greater than most African, Asian, Central and South American, and European nations [1]. Within the US, people who do not self-identify as white and those of lower annual income demonstrate higher geographic mobility, on average, compared to people who self-identify as white and those of greater annual income [2]. Many populations who have traditionally been underrepresented in biomedical research, such as people who are migrant workers, homeless, or identify as gender and sexual minorities, demonstrate exceptionally high rates of geographic mobility.

### 1.2 Overview of the AoURP informed consent process

All persons wishing to participate in AoURP must complete an informed consent process that unambiguously indicates their consent to join. Given its ambitious recruitment goals, the program decided that the primary modality for the consent process would be electronic (i.e., web- or app- mediated) to allow for broad deployment and rapid scaling. Further, it was the program’s desire that the consent process be consistent for all persons regardless of geographic location, enrollment method, or affiliation (participants can enroll directly or through an affiliated healthcare provider organization). Finally, due to the longitudinal and evolving nature of the study and, further, to provide a flexible participant experience, the informed consent for AoURP is modular (Table 1). Following an initial consent experience (Primary Consent), additional “modules” for program activities not included in the Primary Consent can be presented to participants at the program’s choosing and completed by participants at their convenience. At this time, all consent modules require an electronic signature from the participant.

**Figure 1:**
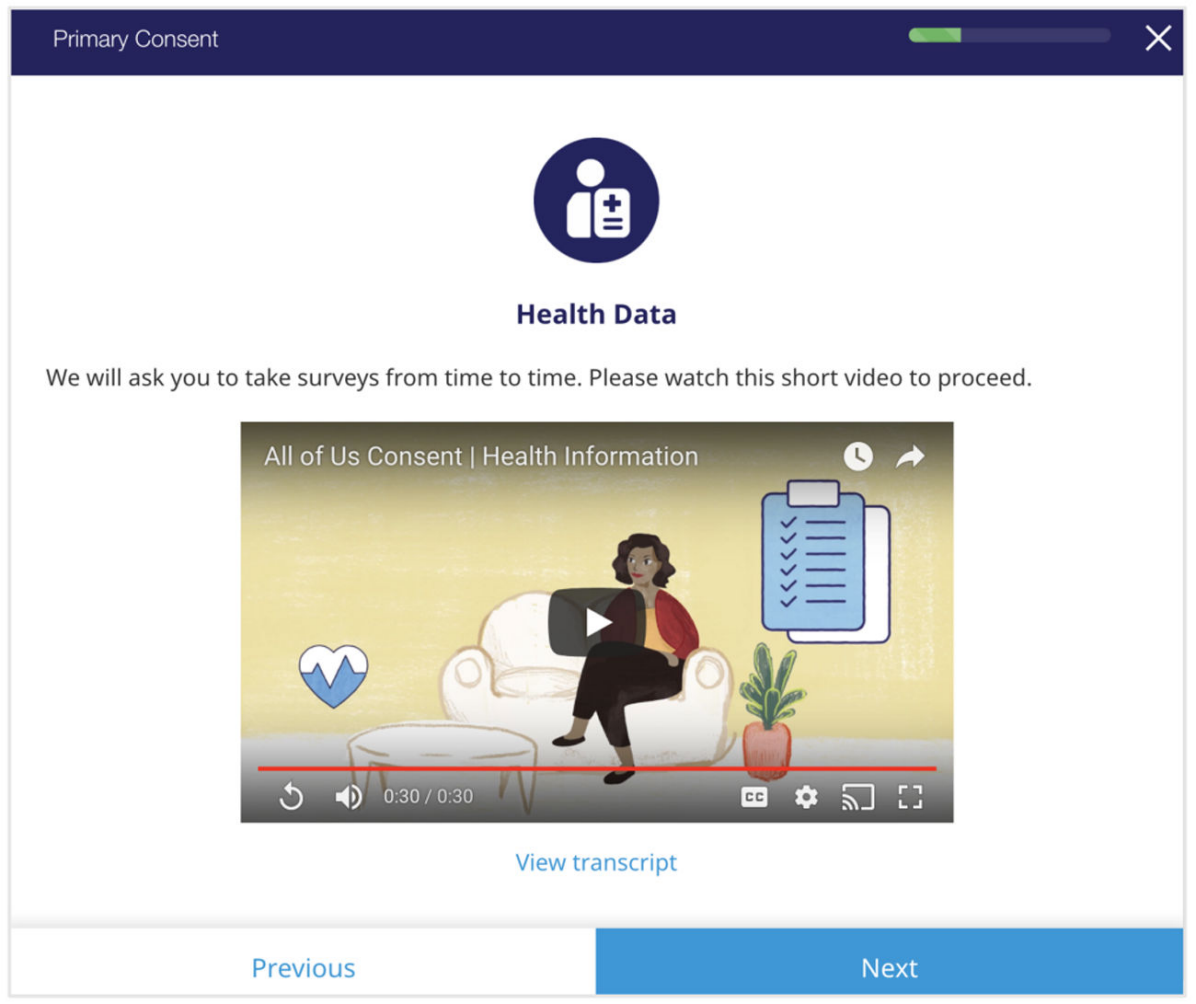
Example AoURP eConsent screen

Each consent module is comprised of three informational components: eConsent screens, formative evaluation questions, and a form requiring signature. The eConsent screens employ visual icons, short videos, and concise, highly structured text blocks to highlight key features of program participation (Figure 1). The formative evaluation is a learning reinforcement tool focusing attention on essential concepts in research participation. Questions specifically target common misconceptions in human subject research (e.g., therapeutic misconception). With the participant’s signature, the form serves as the documentation of participant’s affirmative consent to take part in a given set of research activities.

### 1.3 Choice of law and human subjects research

The AoUPR’s distributed structure and planned duration, when coupled with the geographic mobility of the US population, render questions regarding what research conduct is required, permissible, or prohibited challenging to resolve. Different state and territory laws might apply to the study itself over time and, likewise, to the determination of any given participant’s rights over time. The desired goal of creating a unified informed consent process is further complicated by the threat of vertical conflicts of law (i.e., misalignment of local, state/territory, and federal requirements), horizontal conflicts of law (i.e., differing requirements as a participant moves from state to state or as research efforts are conducted in one location or another), as well as the varying ways in which these conflicts are resolved when disputes arise in tort or contract theory^2^.

A “governing law” or “choice of law” clause allows parties to a contract to specify which jurisdiction’s laws, statutes, and regulations will apply to a contract and be used for dispute resolution and thereby resolve much of the uncertainty or variability in contract interpretation. The US Department of Health and Human Services (HHS) specifies that contracted health services, including human subject research, must include a choice of law clause for work conducted overseas (i.e., outside of the US 50 states, 5 inhabited territories, and District of Columbia) [HHSAR 333.215–70(a)]. By contrast, there is guidance, e.g., the US Food and Drug Administration 21 CFR Part 50.25(d), against choice of law for studies conducted within the US.

While establishing a uniform governing law for the AoURP might be desirable for programmatic ease, this is not easily accomplished. Most injuries from research participation are based in tort theory (not contract theory), and, in tort matters, conflicts of law are typically governed by the rule of *lex loci dilicti* (or the law of the place of the injury). Research consent materials conventionally have not been framed as contracts per se but, rather, as documentation of informed consent or an assumption of risks that would be a full or partial defense to a tort action if one were to arise. Additionally, to the extent consent documents could be construed as contracts, exculpatory language that purports to function as a waiver of participants’ legal rights or a limit on tort liability is generally not permissible (see 21 CFR 50.20). The inclusion of a choice of law provision within informed consent materials has, as a result, not been a viable solution for research in the US. For these reasons, a thorough understanding of state and territory-specific variations in regulations pertaining to human subject research is essential to meeting the program’s regulatory and ethical obligations.

At the US Federal level, informed consent processes for human subject research are guided by the Common Rule [45 CFR Part 46, Subpart A] and overseen by HHS’s Office for Human Research Protections (OHRP). The Common Rule contains a non-preemption clause^3^ as well as direct recognition of additional state-specific informed consent requirements^4^. At least 27^5^ of the 50 states, 5 inhabited territories, and District of Columbia have enacted further jurisdiction- specific regulations regarding human subject research generally, although several simply reference the Common Rule as the guidance standard (Appendix A).

The release of protected health information from covered entities^6^ for research (as well as for other purposes) is regulated by the Health Insurance Portability and Accountability Act of 1996 (HIPAA) Privacy Rule [45 CFR Part 160, Subparts A and E; 45 CFR Part 164] and overseen by HHS’s Office of Civil Rights (OCR). The HIPAA Privacy Rule sets forth a specific set of protections and, for a limited set of enumerated circumstances, allows for state/territory law to offer additional protections^7^ [45 CFR Part 160, Subpart B]. At least 26 of the 50 states, 5 inhabited territories, and District of Columbia have specified further guidance, creating a patchwork of additional regulations across the country (Appendix A).

To enable research regarding substance use disorders to reduce stigma and advance our understanding toward more effective prevention and treatment, AoURP includes records regarding substance use disorder treatment within its request for access to a participant’s protected health information records. In addition to the HIPAA Privacy Rule, release of substance use disorder records is regulated by 42 CFR Part 2, the Confidentiality of Substance Use Disorder Patient Records (Part 2) overseen by HHS’s Substance Abuse and Mental Health Services Administration (SAMHSA). Part 2 details the requirements for release of these records.

Consistent with the core values of AoURP, participants will have access to the full complement of data they contribute to the program. Additionally, with participant consent, the program will interpret a limited set of data for participants; these interpreted data are considered individual research results (IRR). At this time, although the Common Rule applies to IRR equally to all other aspects of human subject research participation, the only Federal law considered by some to be specific to IRR is the Clinical Laboratory Improvement Amendments of 1988 (CLIA) although some have argued the HIPAA Privacy Rule may apply to IRR from non-CLIA certified laboratories [3]. However, at least 17 states’ laws further guide IRR, especially the return of genomic results (Appendix A).

There has been no comprehensive documentation of US state/territory-specific guidance and requirements to date. Therefore, to ensure compliance with all applicable Federal and state/territory guidance and regulation, AoURP consulted with OHRP, OCR, and SAMHSA, sought guidance from the NIH Office of General Counsel, and conducted an independent legal review of the informed consent and HIPAA Authorization processes for this national research program.

## 2. Implementation

We have developed a “parent” version of each consent module. Parent module versions are consistent with the greatest number of state and territory regulations. However, some states and territories have regulations that, if applied to other jurisdictions, might be considered to limit or additionally burden participants. To address these distinctive requirements, we have modified the parent version of modules, creating specific “child” versions of modules for use in those jurisdictions.

### 2.1 State/territory compliant primary consent

To determine the prospective participant’s pathway through the program’s informed consent modules, we ask participants a series of questions. First, we ask participants their state or territory of residence. Those who answer California are presented an Experimental Subject’s Bill of Rights as described by the Protection of Human Subjects in Medical Experimentation Act (California Health and Safety Code 24170–24179.5) in advance of the primary consent. We then ask the participant to confirm they have reached the age of majority for research participation within their state or territory of residence: 18 years of age in all US states and territories with the exception of Alabama (age 19) and Puerto Rico (age 21) (Appendix A). Of note, the Northern Mariana Islands do not have regulations regarding the age of majority; we have elected to use age 18, consistent with the majority of other states and territories. Finally, we ask participants the state or territory in which they receive most of their healthcare.

### 2.2 State/territory compliant HIPAA Authorization/Part 2 data release

We link the version of HIPAA Authorization/Part 2 data release to the state or territory in which the participant reports receiving most of their healthcare. The majority of state- and territory- specific regulations additional to the HIPAA Privacy Rule focus on the term of expiry for the HIPAA Authorization, with states requiring a specific date of expiry or specific term of expiry where the HIPAA Privacy Rule allows for an event of expiry (e.g., the end of the research project). Please see Appendix B for further detail. Of note, the Illinois statute that requires a date of expiry (the Mental Health and Developmental Disabilities Confidentiality Act, 740 ILCS 110), relates only to “therapists.” Therapist is defined as, “a psychiatrist, physician, psychologist, social worker, or nurse providing mental health or developmental disabilities services or any other person not prohibited by law from providing such services or from holding himself out as a therapist if the recipient reasonably believes that such person is permitted to do so”[740 ILCS 110/2 from Ch. 91 1/2, par. 802 section 2], however state convention is to apply this requirement to all Authorizations.

A subset of states require that the release of “sensitive data” such as HIV status, drug and alcohol use, and sexual history be specifically highlighted to the signatory of the release (i.e., MA 104 CMR 31. 05; ORS 192.566; Tex. Bus and Com code 602.051). While the release of these data are highlighted within the parent version of AoURP HIPAA Authorization form to all participants, participants in Massachusetts, Oregon, and Texas are additionally presented with a “sensitive data confirmation” screen as part of the HIPAA Authorization eConsent (Appendix B).

It is important to note that the AoURP HIPAA Authorization does not provide participants the option of granular release of electronic health records; participants either agree to the release of all available records or they decline to give permission for any of their records’ release. This decision was taken by the program based on the program’s core principle of transparency and the technical difficulty of ensuring a completely “clean” data release. We did not want to allow participants the opportunity to request the hold back specific classes of health information only to have that information inadvertently released, for example, within a free-text clinician report about treatment for a separate condition.

Finally, also based on the IL Mental Health and Developmental Disabilities Confidentiality Act, Illinois convention is that HIPAA Authorizations require a “witness signature” in addition to the signature of the participant themselves (Appendix B). The witness can be any person who can attest to the identity of the participant. Interestingly, in Illinois, based on the same statute, withdrawal of consent is also conventionally interpreted to require a witness signature.

### 2.3 State/territory compliant consent for the return of genomic results

AoURP participants may consent to receive medically-actionable genomic testing results, a form of IRR. The specific set of medically actionable results are defined by the program based on professional society guidelines and similar sources (e.g., those of the American College of Medical Genetics and Genomics [4]), and will evolve over time. Given the additional potential risks and benefits the return of medically actionable findings may pose to participants [5], AoURP will use an explicit opt-in informed consent module for the return of genomic results.

Among the relevant state and territory regulations that govern the return of genomic results (Appendix 1), many do not specify if they pertain to clinical care, research endeavors, or both. Further muddying the waters, definitions of genetic information vary [6]. AoURP has elected to use the broad federal definition referenced in the Genetic Information Nondiscrimination Act (GINA) of genetic information which includes family history in addition to information regarding genetic tests [42 U.S.C. § 300gg-91].

Most jurisdiction-specific laws require that the informed consent process for the return of genomic results include a general purpose or description of the genetic tests to be performed, as well as potential uses and limitations of those tests [e.g., Del. Code 16 §1201 (4)]. However, both the State of New York and Commonwealth of Massachusetts require that the consent process include a description, “of each specific disease or condition tested for” [NYCL (CVR) §79- L(2)(b); MGL Public Health 111 §70G(a)]. Notably, NYCL (CVR) §79-L(2)(f) allows for modification of this requirement if, “the research protocol does not permit such degree of specificity.” Additionally, NYCL (CVR) §79-L(9)(a) provides that, “samples may be used for tests other than those for which specific consent has been obtained for purposes of research conducted in accordance with applicable law and regulation and pursuant to a research protocol approved by an institutional review board [IRB] provided that the individuals who provided the samples have given prior written informed consent... and did not specify time limits or other factors that would restrict use of the sample for the test.” Thus, a broad description of the diseases or conditions tested for is allowed under IRB oversight for participants within the State of New York.

In the case of Massachusetts, there is no explicit clause within MGL Public Health 111 §70G that specifies any ability to modify the requirement for inclusion of a general description of each specific disease or condition tested for within the consent process. However, current research convention mirrors New York’s: with the oversight of an IRB, participants of genomic research are consented to the return of genomic results of broad description. In practice, both the AoURP parent eConsent and consent form for the return of genomic results will link out to an inventory of conditions being tested for with explicit notation that this list may be updated over time. Additionally, in consideration of subpart (c) of the Massachusetts statue, this inventory will address for all participants each tests’ reliability and predictive value.

Massachusetts further specifics a discussion with, “the medical practitioner ordering the test” regarding the reliability and certainty of test results prior to consent. Given the research context of AoURP’s return of genomic results, genetic counseling will be made available to all participants prior to completing the consent process, regardless of their state of residence, but will not be required. This is also consistent current practice in Massachusetts.

In FLA. Stat. Ann 760.40(3), the State of Florida sets forth a number of requirements for DNA analysis and the return of results.^8^ Two of these requirements are incorporated into the parent version of the return of genomic results consent process for all AoURP participants. First, AoURP will enable participants to track the journey of their sample from receipt by the biobank, to analysis for tests specified in the return of genomic results inventory, to its receipt by the genetic counseling core and/or deposit in their AoURP participant record. Secondly, the parent consent form includes a statement that AoURP, as a research program, is not engaged in any decisions to grant or deny insurance, employment, mortgage, loan, credit, or educational opportunities and, therefore, that these results will not be used for those purposes by the program.

The one FLA. Stat. Ann 760.40(3)-required customization of the return of genomic results consent process not incorporated into the parent consent process will be accommodated by an addition to the eConsent (Appendix B). Within the eConsent process, residents of the state of Florida will be able to specify a healthcare provider to whom the participant would like their results sent [FL 760.40 (3)]. This feature will likely be made available to all participants (once trialed in Florida), pending review of relevant state-specific considerations. In the interim, study participants may independently choose to share their test results with healthcare providers.

## 3. Conclusion

The *All of Us* Research Program is an ambitious national cohort study designed to accelerate understanding of human health. The diversity of laws, statutes, and regulations across the US challenge large, dispersed research efforts such as AoURP in ways not unlike those faced by international research efforts [7]. Creating a pattern of distinct informed consent interactions over time, with each consent module having its own specific ask, including potential risks, benefits, and set of scientific “unknowns” that arise naturally in cutting edge research, supports participant autonomy while allowing for flexibility in the face of legal and regulatory uncertainty. Empirical legal research will be essential to facilitate this and similar biomedical research efforts and to enable research teams in their efforts to respect and promote participant’s rights.

There are several limits to our analysis. First and foremost, despite having consulted with experts across the nation, there is no central clearinghouse or curated resource for the most current US state/territory research regulations. Additionally, as we noted in our analysis, it is sometimes difficult to tease apart state/territory requirements and convention. As the clinical and research genetics community knows well, few of these rules and regulations have been adequately stress- tested in the courtroom, leaving a dearth of guidance for researchers and policy makers alike. It is also important to note that while this analysis is, to the best of our knowledge, complete as of January 1, 2018, laws, technologies, research practices, and societal norms are constantly evolving; AoURP will engage in regular re-review of its consent materials and approaches to ensure their currency.

## Figures and Tables

**Table 1:** Overview of AoURP consent modules

Module	Addresses
**Primary**	Overview of all program activities. Signature indicates consent to take part in surveys and data linkage from external sources (e.g., state cancer registries), and, if invited, physical measurements, biospecimen collection (including biobanking and biomarker/genomic assays), and sensor/wearable technology activities.
**HTPAA Authorization**	Signature indicates consent to regular collection of electronic health records from all identifiable health care providers/entities including Part 2 (substance use disorder treatment) records and personally identifiable information (PII) from any source.
**Return of Genomic Results**	Signature indicates consent to receive medically-actionable genomic testing results from the program.

## Footnotes

2 Conflicts of law are resolved by courts in a number of ways, including use of the *lex loci dilicti* rule, “most significant contacts” test, “comparative governmental interest” test, or a combination.

3 “This policy does not affect any State or local laws or regulations which may otherwise be applicable and which provide additional protections for human subjects”

4 “The informed consent requirements in this policy are not intended to preempt any applicable Federal, State, or local laws which require additional information to be disclosed in order for informed consent to be legally effective.”

5 This count includes regulations specific to HIV testing and status, as well as general regulations

6 Defined by 45 CFR § 160.103 as “(1) A health plan. (2) A health care clearinghouse. (3) A health care provider who transmits any health information in electronic form in connection with a transaction covered by this subchapter.”

7 When “State law has the specific purpose of protecting the privacy of health information or affects the privacy of health information in a direct, clear, and substantial way” (i.e., the state/territory law “relates to the privacy of individually identifiable health information” as defined by HIPAA at 45 CFR 160.202) and “is more stringent” than HIPAA (as “more stringent” is defined by HIPAA at 45 CFR 160.202).

8 FLA. Stat. Ann 760.40(3), a civil rights statute which predates GINA, was drafted to prevent “surreptitious,” and potentially discriminatory, genetic testing without a focus on the statues’ potential implications for research. For this reason, IRBs in Florida have generally rejected a narrow interpretation of this statute when considering research initiatives like AoURP.

## 5. Appendices

### Appendix A: State/territory laws informing the *All of Us* Research Program primary informed consent process, HIPAA Authorization, and Return of Genomic Results consent

**Table:**

Domain	State	Statue
**Age of Majority**	Alabama	Ala. Code § 26-1-1- Infants and incompetents Although not germane to AoURP, nb subpart (f), “a person who is 18 years of age or older may consent to participate in research conducted by a college or university that is accredited by a federally recognized accrediting agency if the research has been approved by the Institutional Review Board of the institution.”
	Puerto Rico	31 L.P.R.A. §971
**Bill of Rights**	California	California Health and Safety Code 24170-24179.5
**Primary Consent**	Alabama	AL Code § 22-56-4 AL Code § 22-11A-51; § 22-11A-53
	Arizona	AZ Rev Stat § 36-663
	California	Cal Health & Safety Code § 24173; Cal Pen Code § 3521 CA Health & Safety Code § 121075; § 121105
	Colorado	Col Rev Stat § 25-4-410
	Connecticut	CT Gen Stat § 19a-583; § 19a-585; § 19a-582
	Delaware	16 DE Code § 715
	District of Columbia	DC Code § 7-1305.09
	Guam	Ch 24 Guam Research Review Board § 24106
	Hawaii	HI Rev Stat § 325-16
	Illinois	Illinois Mental Health and Developmental Disabilities Confidentiality Act; AIDS Confidentiality Act 410 ILCS 50/3.1 410 ILCS 305/8
	Kansas	KS Code § 65-4974
	Massachusetts	104 CMR 31.05 ALM GL ch. 111, § 70F
	Montana	TITLE 53. SOCIAL SERVICES AND INSTITUTIONS CHAPTER 21. MENTALLY ILL 53-21-147
	Nebraska	NE Rev Stat § 71-531
	New Hampshire	NH Rev Stat 141-F:5
	New Jersey	NJ Stat. § 26:14-4; N.J. Stat. § 26:14-5
	New Mexico	NM Stat § 24-2B-2
	New York	NY CLS Pub Health § 2441; NY CLS Pub Health § 2442 NY CLS Pub Health § 2781; NY CLS Pub Health § 2782
	Oklahoma	63 OK Stat § 63-3102A
	Oregon	ORS 433.075
	Pennsylvania	35 P.S.§ 7605
	South Carolina	SC Code § 44-26-180
	South Dakota	SD Codified L § 27B-8-41.
	Texas	Texas Health & Safety Code § 81.105; §81.106.
	Virginia	VA Code Ann. § 32.1-162.20; § 32.1-162.16; § 32.1-162.18
	Washington	RCW § 70.24.330
	West Virginia	WV Code § 16-3C-2
**HTPAA Authorization**	Alabama	AL Code § 22-11A-22; AL Code § 22-11A-54
	Arizona	AZ Rev. Stat. § 36-664
	California	CA Civ Code § 56.10
	Colorado	CO Rev Stat § 25-1-1201
	Connecticut	CT Insurance Information and Privacy Protection Act § 19a-581; § 19a-585; Conn. Gen. Stat. § 20-7c; Conn. Gen. Stat. § 52-146g
	Delaware	Del. Code Ann. tit. 16, § 717; Del. Code § 1212
	District of Columbia	DC Code § 7-1605; § 7-1203.06
	Florida	FL Stat § 381.004
	Georgia	GA Code § 24-12-2; § 24-12-21; § 24-12-12; § 31-33-8; § 37-4-125
	Hawaii	HI Rev Stat Ann § 325-101
	Illinois	Personal Information Protection Act § 50; 735 ILCS 5/8-2001
	Indiana	IN Code § 16-39-2-5
	Iowa	Iowa Code § 228.2; § 228.3; § 228.4; § 141A.9
	Louisiana	LA Rev Stat. § 22:1023
	Maine	ME Rev Stat § 1711-C
	Maryland	MD HEALTH-GENERAL Code Ann. § 4-303
	Minnesota	MN Stat § 144.293;144.294;144.295
	Montana	MT Code § 50-16-502; § 50-16-527; § 50-16-1009
	New Mexico	NM Stat § 24-2B-6; § 24-2B-7; § 43-1-19; § 24-14B-6
	Ohio	OH Rev Code § 3701.17;§ 3701.243; § 5119.27
	Oklahoma	OK Stat §43A-1-109
	Oregon	OR Rev Stat § 192.553; § 192.556; §192.566; § 431A.865
	Pennsylvania	Title 35 P.S. Health and Safety § 7607
	Puerto Rico	Title 26 Subtitle 3 Chapter 112 § 9240
	Rhode Island	RI Gen L § 5-37.3-4
	Texas	INS § 602.051
**Return of genomic results** ^9^	Alaska	AS §18.13.010
	Delaware	Del. Code 16 §1201 et seq.
	Florida	FS §760.40(2)(a); FS §760.40(3)
	Georgia	OCGA §33-54-3(b)
	Iowa	Iowa Code §§729.6
	Massachusetts	MGL Public Health 111 §70G(a)
	Michigan	MCL §333.17520(2)
	Minnesota	MS §13.386 Subd.3(a)
	Nebraska	NRS §71-551(1)
	Nevada	NRS §629.151; §629.161; §629.181; §629.101 et seq.
	New Hampshire	NHS §141-H:1; NHS § 141-H:2
	New Jersey	NJ Rev Stat §10:5-45
	New Mexico	NMSA §24-21-3
	New York	NYCL (CVR) §79-L(2)(b); NYCL (CVR) §79-L(9)(c); NYCL (CVR) §79-L(9)(e)
	Oregon	ORS §192.535; ORS §192.538(5)
	South Carolina	SCCL §38-93 et seq.
	South Dakota	SDCL §34-14-22

### Appendix B: Summary of State-Specific Variations of the AoURP Consent Process

**Table:**

States/Territories	Primary Consent			HIPAA Authorization		Return of Genomic Results Consent	
	*Bill of* *Rights*	*eConsent* *version*	*Form* *version*	*eConsent* *version*	*Form* *version*	*eConsent* *version*	*Form* *version*
AL, AK, AZ, AR, CO, CT*, DC, GA*, HI, ID, IA, KS, KT, MI, MS, MO, NE**, NV, NH, NJ, NM, NY, NC, ND, PA, RI, SC, SD, TN, UT, VT, VA**, WV, WI, Puerto Rico, US Virgin Islands, Guam, American Samoa, Northern Mariana Islands	none	Parent	Parent	Parent	Parent	Parent	Parent
CA	required	Parent	Parent	Parent	Date of expiry: Standard	Parent	Parent
DL, IN, LA, MN, OH, OK, WA	none	Parent	Parent	Parent	Date of expiry: Standard	Parent	Parent
MA, OR, TX	none	Parent	Parent	Sensitive Data Confirmation	Parent	Parent	Parent
ME, MT**	none	Parent	Parent	Parent	Date of expiry: 30-month	Parent	Parent
MD, WY	none	Parent	Parent	Parent	Date of expiry: 12-month	Parent	Parent
IL	none	Parent	Parent	Witness Signature	Access to records	Parent	Parent
FL	none	Parent	Parent	Parent	Parent	Share with healthcare provider	Parent

* In Connecticut and Georgia HIPAA Authorizations are valid for one year from their date of signature to request of records from insurance providers.

** In Montana, Nebraska, and Virginia HIPAA Authorizations are valid for two years from their date of signature for the request of records from insurance providers.

Other notes:
In the states of Maine and Montana, HIPAA Authorizations are only valid 30 months (in Montana, only if expiry date is provided). Given the nature of rolling enrollment, we will update the form used by those in Maine and Montana on an annual basis to state a date 30 months from January 1st of the enrollment year. At the date of expiry (30 months from January 1st of the enrollment year), all persons consented that calendar year would be contacted for re-authorization on a form listing a date 30 months hence. For example, the 2018 form will expire on 7/1/2020. Those consented in 2018 would be asked to re-sign a form 7/1/2020 expiring 12/31/2022. In the states of Maryland and Wyoming, HIPAA Authorizations are only valid for one year. Annually, people who receive care in Maryland or Wyoming will be invited to re-sign the same form with a 12-month expiry (but no date) listed.
